# The relationship between reward and punishment processing and the 5-HT_1A_ receptor as shown by PET

**DOI:** 10.1007/s00213-013-3426-9

**Published:** 2014-01-16

**Authors:** Paul Faulkner, Sudhakar Selvaraj, Alex Pine, Oliver D. Howes, Jonathan P. Roiser

**Affiliations:** 1 Institute of Cognitive Neuroscience, University College London, 17 Queen Square, London, WC1N 3AR UK; 2 Medical Research Council Clinical Sciences Centre, Hammersmith Hospital, London, W12 0NN UK; 3 Department of Neurobiology, The Weizmann Institute of Science, Rehovot, 76100 Israel

**Keywords:** Serotonin, Positron emission tomography (PET), Decision-making, 5-HT_1A_ receptor, Cognition, Hippocampus

## Abstract

**Rationale:**

The serotonin (5-HT) system has been reported to be involved in decision-making. A key component of this neurotransmitter system is the 5-HT_1A_ receptor, and research is beginning to show how this receptor can influence decision-making. However, this relationship has rarely been studied in humans.

**Objectives:**

This study assessed whether individual variability in 5-HT_1A_ availability correlates with decision-making in healthy volunteers.

**Methods:**

We measured regional availability of the 5-HT_1A_ receptor in the hippocampal complex and striatum using positron emission tomography and correlated this with performance on two decision-making tasks measuring sensitivity to probability, rewards and punishments and temporal discounting, respectively.

**Results:**

No relationship between decision-making behaviour and 5-HT_1A_ availability in the striatum was found. However, a positive correlation was detected between participants’ 5-HT_1A_ availability in the hippocampal complex and their sensitivity to the probability of winning. Furthermore, there was a negative correlation between the degree to which participants discounted future rewards and 5-HT_1A_ availability in the hippocampal complex.

**Conclusions:**

These data support a role for the 5-HT_1A_ receptor in the aberrant decision-making that can occur in neuropsychiatric disorders such as depression.

**Electronic supplementary material:**

The online version of this article (doi:10.1007/s00213-013-3426-9) contains supplementary material, which is available to authorized users.

## Introduction

The serotonin (5-HT) system has been implicated in decision-making, with manipulations resulting in altered processing of rewards (Murphy et al 2002) and punishments (Cools et al 2008; Crockett et al 2009). Specifically, reduced 5-HT transmission has been implicated in impulsivity (Winstanley et al. 2004), a component of which is temporal discounting, the tendency to devalue temporally distant rewards. Experimentally increasing levels of 5-HT in rats via administration of an SSRI decreases discounting behaviours (Bizot et al 1988), whereas reducing 5-HT in humans via administration of acute tryptophan depletion (ATD) increases discounting (Schweighofer et al 2008). Interestingly, Takahashi et al (2008) reported that depressed patients, in whom 5-HT transmission is hypothesized to be disrupted, also exhibited elevated temporal discounting.

The striatum is known to play a central role in decision-making (Cools et al 2007). Schultz et al. (2000) demonstrated that neurons within the primate striatum fired in response to reward-predicting stimuli, while in humans Delgado et al. (2000) reported that the dorsal striatum was active during monetary reward processing. With respect to impulsivity, Pine et al. (2009) showed that delay discounting correlated with responses in the human striatum. 5-HT transmission in the striatum may also influence decision-making: Seymour et al. (2012) found that ATD altered the exchange rate by which rewards and punishments were compared, which was linked to an increase in striatal and prefrontal responses. Moreover, Tanaka et al. (2007) observed that the ventral striatum responded to short-term rewards after ATD, while the dorsal striatum responded to long-term rewards after acute tryptophan loading.

The hippocampus may also play a role in decision-making: Lisman and Grace (2005) argue that the hippocampus encodes novel information, with such novelty signals being conveyed to the VTA via the ventral striatum, which means that these signals may regulate striatal-dependent reward processing. Indeed Camara et al. (2008) showed increased striatal–hippocampal coupling during the processing of gains and losses in humans, and Guitart-Masip et al. (2010) reported that novel images (which activated the hippocampus) enhanced reward signals in the striatum elicited due to subsequently presented rewards. Regarding impulsivity, Mariano et al. (2009) reported that rats with hippocampal damage exhibited increased discounting of delayed rewards, which Schacter and Addis (2009) argue is due to the hippocampal complex being involved in the formation of past and future episodic representations. Similarly, Peters and Büchel (2010) suggest that temporal discounting is related to the subjective capacity for future episodic thought, with such ‘mental time travel’ involving the hippocampus. The hippocampus’ role in decision-making may also depend on 5-HT transmission: ATD results in poorer performance on episodic memory tasks (Riedel et al 1999), argued to be due to a decreased stimulation of excitatory 5-HT receptors (Meeter et al 2006). Mobini et al. (2000) reported that rats whose 5-HT systems had been destroyed with 5,7-dihydroxytryptamine became more impulsive, choosing smaller, sooner rewards, which correlated with the magnitude of 5-HT depletion in the hippocampus. Initial evidence implicates the 5-HT_1A_ receptor in decision-making: Schmitz et al. (2009) showed that human subjects homozygous for the 5-HT_1A_ C(-1019)G polymorphism, linked to increased 5-HT_1A_ receptor expression, exhibited longer reaction times to potential rewards and shorter reaction times to potential punishments. With respect to temporal discounting, Miyazaki et al. (2012) demonstrated that injection of the 5-HT_1A_ receptor agonist 8-hydroxy-2-(di-*n*-propylamino)-tetralin into the rat dorsal raphe nucleus, which decreases 5-HT neuron firing rates, increased waiting errors for delayed but not immediate rewards.

Whilst not specifically relating to the 5-HT_1A_ receptor, recent work by Macoveanu and colleagues has implicated another 5-HT receptor sub-type in decision-making: Macoveanu et al. (2013b) report that acute blockade of the 5-HT_2A_ receptors with ketanserin made healthy volunteers more risk-averse and selectively reduced ventral striatal responses to negative outcomes. Further, ketanserin abolished the negative correlation between striatal responses to low-risk negative outcomes and risk-averse choice behaviour that was observed in the placebo condition. Using multi-modal neuroimaging, the same group also demonstrated that lateral prefrontal activation during response inhibition was attenuated following tryptophan depletion and that the magnitude of this reduction was greatest in those with high cortical 5-HT_2A_ binding (Macoveanu et al. 2013a). Together these two studies indicate the ability of neuroimaging methods in helping researchers to better understand the relationship between decision-making and activity at a specific 5-HT receptor sub-type.

While 5-HT transmission in the striatum and hippocampus, particularly at the 5-HT_1A_ receptor, may play an important role in decision-making, this hypothesis is difficult to test in humans as regionally specific pharmacological manipulations cannot be conducted non-invasively. To address this question, we utilized an individual differences approach, examining whether a relationship between decision-making and 5-HT transmission could be observed at the level of the receptor. Whilst this approach was used to demonstrate that dopamine transmission influences decision-making (Takahashi 2012), it has never been used to better understand 5-HT’s influence on decision-making (Takahashi et al 2010). We measured levels of the 5-HT_1A_ receptor using positron emission tomography (PET) and correlated these with performance on two decision-making tasks, measuring sensitivity to reward and punishment information, and temporal discounting, respectively. We predicted that greater 5-HT_1A_ availability in the striatum and hippocampus would predict increased sensitivity to rewards and punishments and decreased temporal discounting.

## Materials and methods

### Participants

Fifteen participants (13 males; mean age 50.9 years, range 35–63 years) underwent a PET scan at the Cyclotron Unit at the Hammersmith Hospital campus of Imperial College London and completed a separate behavioural session on a different day at the Institute of Cognitive Neuroscience, University College London. Subjects were free from past or present psychiatric disorders, as determined by assessment on the Mini International Neuropsychiatric Inventory (MINI; Sheehan et al 1998). None of the subjects had taken any psychotropic medication within 12 months of participating nor had they any previous history of alcohol/substance dependence. Written informed consent was obtained from all subjects prior to both scan and behavioural sessions, and the study was approved by the local research ethics committee. At the behavioural session, participants received £20 for compensation for their time and travel, and up to an additional £10 on each of the two cognitive tasks, depending on performance, meaning that each volunteer was compensated between £20 and £40 for this session.

### PET procedure

To measure regional 5-HT_1A_ availability, we used ^11^C-CUMI-101, a highly selective 5-HT_1A_ PET ligand (Milak et al 2011). A full description of PET data acquisition and analysis methods is presented in Selvaraj et al. (2012). Briefly, participants had an intravenous canula sited in the ante-cubital vein flushed with a saline infusion followed by an injection of the ^11^C-CUMI ligand as a smooth bolus over a 30-s period at the beginning of the scan. The radiochemical purity of the ligand was high (between 95 % and 100 %, mean 97 %, SD 1.5 %).

PET scans were acquired from a GE Discovery RX PET/CT scanner with an axial field view of 15.7 cm. Twenty-two frames were acquired in total, each with 47 slices at 3 mm in thickness. The dynamic PET scans were acquired over 90 min. Time frames were of increasing duration: 30-s pre-injection background, 1 × 15 s, 3 × 5 s, 1 × 30 s, 4 × 60 s, 7 × 300 s, and 5 × 600 s. The dynamic scans were de-noised using a level 2, order 64 Battle Lemarie wavelet filter (Turkheimer et al 1999). Head movement in the dynamic PET acquisition was corrected for using frame-by-frame realignment using a mutual information algorithm (Studholme et al 1997).

For our analyses, we calculated whole-brain parametric images in order to assess relationships between 5-HT_1A_ availability and behavioural performance on a voxel-wise basis. These were acquired from the dynamic images using Piwave software (Turkheimer et al. 2007) with the simplified reference tissue model method (Lammertsma and Hume 1996). Each subject’s parametric image was spatially normalised to the PET template within SPM8 software (statistical parametric mapping 8; www.fil.ion.ac.uk/spm/software/spm8/) and smoothed with an 8-mm full-width at half-maximum Gaussian kernel. The ROIs of the hippocampal complex (hippocampus and parahippocampal gyrus combined) and the striatum (nucleus accumbens, putamen and pallidum, due to very low binding values within the caudate) were defined using the HamNet probabilistic atlas (shown to have a very high reliability for these regions; Hammers et al 2007).

### Behavioural session

#### Gambling task (Rogers et al. 2003)

**Fig. 1:**
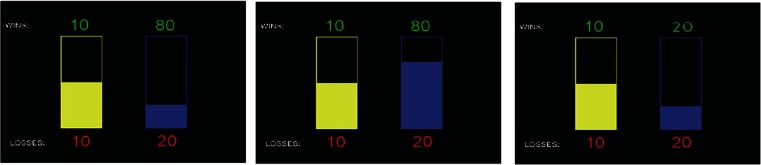
Example trials from the gambling task showing the ‘control’ gamble (*left*) consisting of a 50 % chance of winning or losing ten points and the ‘experimental’ gamble (*right*). Possible wins are in *green*, and possible losses are in *red*

Participants completed 80 trials, each of which required them to make a choice between two gambles. They were paid according to their winnings, with each point won being converted to one penny. Each gamble was represented as a bar, the height of which conveyed the probability (25, 50 or 75 %) of winning or losing a number of points. The sizes of potential gains and losses were displayed in green at the top and in red at the bottom of the bars, respectively (Fig. 1).

On each trial, participants had to choose between the ‘control’ gamble, which consisted of a 50 % chance of winning or losing ten points, and an ‘experimental’ gamble, which varied in terms of probability, high (75 %) or low (25 %), potential gains, high (80 points) or low (20 points) and potential losses high (80 points) or low (20 points), resulting in eight trial types. The gambling task also included two further trial types (gains only and losses only), which were not analysed further.

This task yields three main outcome measures: the proportion of choices of the experimental gamble over the control gamble as a function of (1) probability of winning, (2) the size of potential gains and (3) the size of potential losses. These measures were calculated by taking the difference between the proportion of experimental gamble chosen when each of these factors was high compared with when they were low (for clarity, the negative of this value is presented for loss sensitivity).

#### Temporal discounting task (Pine et al. 2009)

**Fig. 2:**
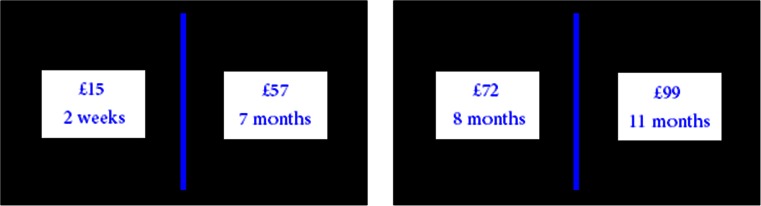
Subjects had to choose between a smaller, more immediate reward (*left side* of *blue bar*) and a larger but more delayed reward (*right side*). The amounts of money differed in magnitude (£1 to £100) and in delay (1 week to 1 year). Subjects completed 220 trials, giving them different scenarios each time

Participants completed 220 trials. In 200 of these trials, the subjects chose between two scenarios, one in which they would receive a smaller amount of money in the more immediate future and one in which they would receive a larger amount of money in the more distant future (Fig. 2). The remaining 20 trials were ‘catch’ trials, where participants could choose between receiving a smaller amount of money after a longer time delay, or a larger, sooner amount. This trial type was administered to ensure that participants were engaged with the task. The choices varied in magnitude (from £1 to £100) and delay (from 1 week to 1 year).

Values were assigned to each option within each trial, implemented in the context of a hyperbolic discount valuation model taken from Mazur (1987) and developed further by Pine et al. (2009). Critical to subjects’ choices in this task is the steepness of the discounting of expected reward values according to their delay and magnitude, denoted by:

$$ \begin{array}{c}\hfill V=D(d)*U(M)=\frac{1\ {e}^{\left(- rM\right)}}{r\left(1+K*d\right)}\hfill \\ {}\hfill D=\frac{1}{1+ Kd}\hfill \\ {}\hfill U=\frac{1-{e}^{\left(- rM\right)}}{r}\hfill \end{array} $$

where *V* is the subjective value (or ‘subjective discounted utility’) placed upon an expected reward of a magnitude *M* with a delay of *d. D* is the discount factor (ranging from 0 to 1) by which the utility (*U*) is discounted, being a hyperbolic function of the delay of the reward multiplied by the discount rate parameter *K*. A higher *K* value indicates a higher rate of discounting, while a ‘flat’ discounting rate (*K* = 0) implies no discounting at all, choosing the more delayed reward, no matter how long the wait. Finally, *U* is a negative exponential function of the magnitude of the reward, including a parameter, *r*, which describes the concavity/convexity of the participant’s utility function and hence their sensitivity to increasing magnitudes of reward. In addition to the number of smaller, sooner vs. larger, later options chosen, participants’ *K* and *r* values were the main outcome measures, determined using maximum likelihood estimation (see Pine et al. (2009) for details).

### Analysis

Participants’ scores from each of the behavioural tasks were entered as group-level covariates in second-level SPM8 analyses including their whole-brain parametric PET images to identify correlations between behaviour and regional 5-HT_1A_ availability. Each variable from each task was entered separately, resulting in five second-level models (win sensitivity, loss sensitivity and probability sensitivity from the gambling task, and discount rate (*K*) and utility concavity (*r*) from the temporal discounting task). We corrected for multiple comparisons, controlling the family-wise error rate at the voxel level. We were particularly interested in relationships between 5-HT_1A_ availability and behaviour in striatal and hippocampal regions. Therefore, we used the hippocampal and striatal ROIs (see prior text) in order to constrain the search volume, resulting in small volume corrected (SVC) P-values. However, due to the exploratory nature of this study, we did not correct over the volume of merged ROIs but instead examined each (bilateral) ROI separately in order to ensure that any significant correlations with 5-HT_1A_ availability were observed.

## Results

### Gambling task

Subjects chose the ‘experimental’ gamble significantly more often when the probability of winning was higher (*t*[14] = 15.0, *P* < 0.001), when the amount they could win was higher (*t*[14] = 2.9, *P* = 0.011) and when the amount they could lose was lower (*t*[14] = 3.0, *P* = 0.009). The mean sensitivity to win was 0.110 (SD = 0.147), the mean sensitivity to losses was 0.115 (SD = 0.146) and the mean sensitivity to probability was 0.694 (SD = 0.18). Sensitivities to wins and losses were strongly positively correlated (*r* = 0.722, *P* = 0.002).

**Fig. 3:**
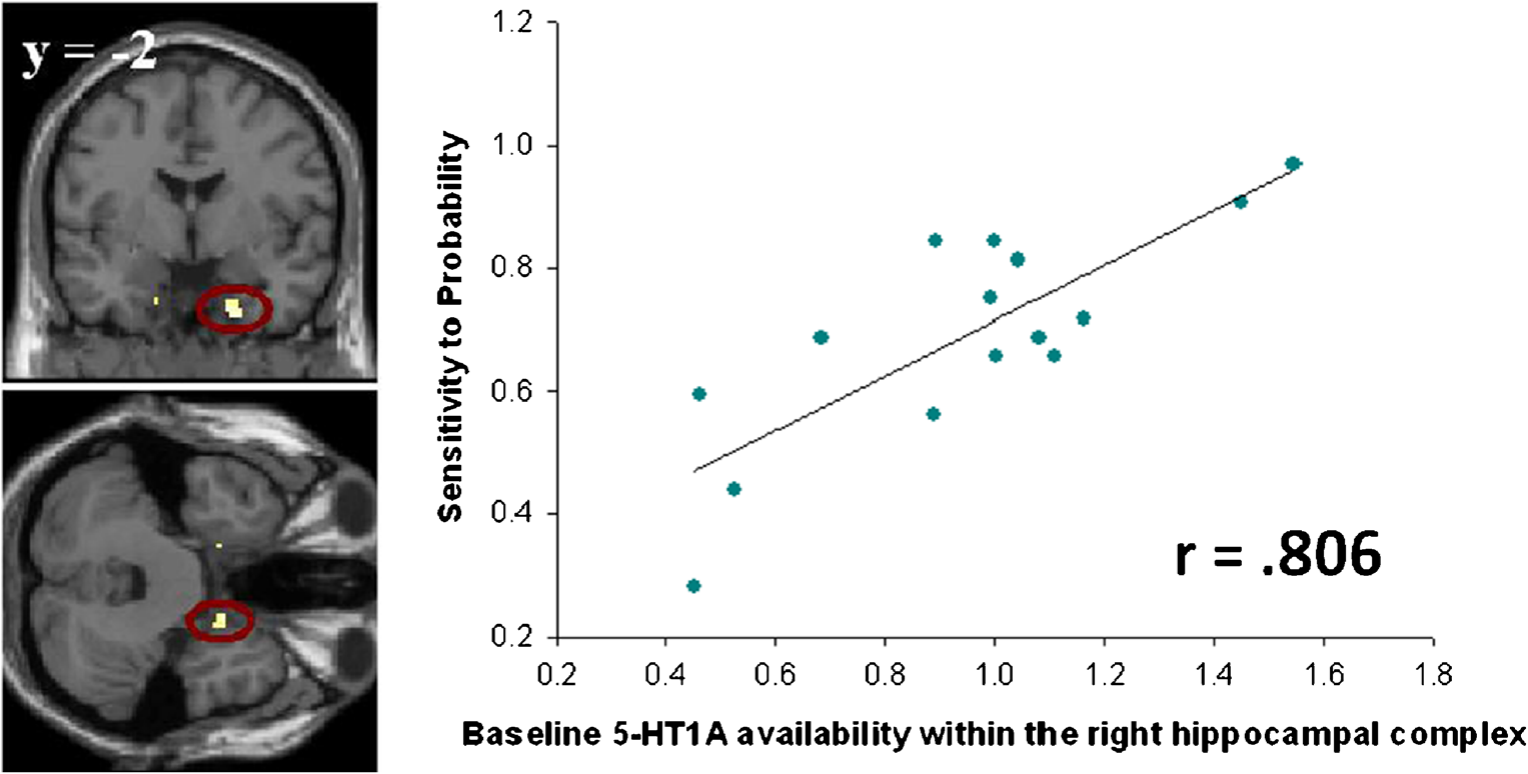
*Left* statistical parametric map (SPM) depicting ^11^C-CUMI binding at baseline within the right hippocampal/parahippocampal ROI that positively correlates with participants’ sensitivity to probability (20, 0, −38, small volume correction analysis presented at *P*_SVC_ = 0.028). *Right* scatterplot of correlation between participants’ sensitivity to probability and baseline ^11^C-CUMI binding within this cluster. This correlation shows that those participants who showed greater sensitivity to information pertaining to probability had greater baseline 5-HT_1A_ binding within the right hippocampal complex

In the voxel-wise analyses, positive correlations were identified between sensitivity to probability and 5-HT_1A_ availability in the hippocampal complex bilaterally, though only the correlation in the right survived SVC correction for multiple comparisons (right peak voxel: [*x* = 20, *y* = 0, *z* = −38], *Z* = 3.63, *P*_SVC_ = 0.028 (Fig. 3); left peak voxel: [*x* = −16, *y* = −2, *z* = −34], *Z* = 3.21, *P*_SVC_ = 0.089). This correlation between 5-HT_1A_ availability in the right hippocampal complex and sensitivity to probability remained significant, surviving SVC correction for multiple comparisons, even when the two female participants were excluded from the analysis ([*x* = 20, *y* = 0, z = −36], *Z* = 3.51, *P*_SVC_ = 0.045). There was no significant relationship between 5-HT_1A_ availability in the striatum and any of the parameters from the gambling task. For completeness, we provide a list of all regions surviving a threshold of *P* < 0.001 (uncorrected), minimum cluster size of 10 voxels, in Table 1 of the “Electronic supplementary material”.

### Temporal discounting task

All subjects chose the larger–sooner reward on above 90 % of the catch trials (mean 19.4/20, SD = 0.83). On average, participants chose the sooner, smaller option over the larger, greater option (mean 119.2/200, SD = 48.05).

Using the model of best fit from Pine et al. (2009), we found that participants discounted the value of future rewards hyperbolically (mean *K* = 0.099, SD = 0.057) and also exhibited a concave utility function (mean *r* = 0.0086, SD = 0.017), comparable to results reported previously on this task. Participants’ *K* values and *r* values were significantly correlated (*r* = 0.565, P = 0.028).

**Fig. 4:**
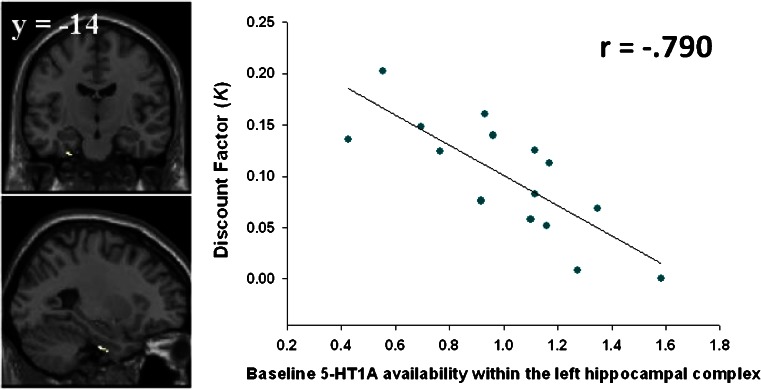
*Left* SPM image depicting baseline ^11^C-CUMI binding within the left hippocampal/parahippocampal ROI that negatively correlates with participants’ discount factor (−26, −14, −36, small volume correction analysis presented at *P*_SVC_ = 0.037). *Right* scatterplot of correlation between discount factor and baseline 5-HT_1A_ receptor availability within this cluster. This correlation indicates that those participants who displayed increased discounting of rewards based upon their temporal delay had decreased baseline 5-HT_1A_ receptor binding within the left hippocampal complex

The voxel-wise analyses revealed significant negative correlations between *K* and 5-HT_1A_ availability in the left hippocampal complex (Fig. 4) which survived correction for multiple comparisons (peak voxel: [*x* = −26, *y* = −14, *z* = −36], *Z* = 3.54, *P*_SVC_ = 0.037). This correlation however did not remain significant when removing the two female participants from the analysis. Again there was no significant relationship between 5-HT_1A_ availability in the striatum and either *K* or *r* values. A full list of all regions that survive a threshold of *P* < 0.001 (uncorrected), minimum cluster size of 10 voxels, is presented in Table 2 of the “Electronic supplementary material”.

## Discussion

Several studies have suggested that 5-HT transmission in the striatum and hippocampus may play an important role in decision-making and impulsivity. Using an individual differences approach, we identified a positive correlation between participants’ sensitivity to probability on a gambling task and the 5-HT_1A_ availability in the hippocampal complex, and a negative correlation between participants’ discount rate on the temporal discounting paradigm and 5-HT_1A_ availability in the left hippocampal complex. However, no such relationships were found between performance on these tasks and 5-HT_1A_ availability in the striatum.

The involvement of the hippocampal complex in participants’ sensitivity to probability supports previous research that has highlighted a role for this structure in reward and punishment processing (e.g. Camara et al 2008). Furthermore, it supports the notion that 5-HT may have a role to play in this relationship since those participants with greater 5-HT_1A_ availability were more likely to use information about the probability of winning when making their decisions on the gambling task. This finding is consistent with that of Rogers et al. (1999), who showed that ATD resulted in a reduced selection of more probable gain outcomes. No such relationship was seen with regards to the amount that participants use information about the magnitude of potential wins or losses. This may be due in part to actual win, loss and probability sensitivity scores of 0.110, 0.115 and 0.694, respectively. These scores differ from those reported in a previous study using the same task where ATD reduced win sensitivity (Rogers et al 2003), which were 0.30, 0.25 and 0.52, respectively. In other words, participants in the present study tended to rely more strongly on information regarding the probability of an outcome rather than the outcome itself, which may have affected our sensitivity to identify correlations with gain and loss sensitivity measures.

The involvement of hippocampal complex 5-HT_1A_ availability in participants’ intertemporal choices is consistent with previous research that has highlighted its role in temporal discounting (e.g. Mobini et al 2000; Schacter and Addis 2009) and previous work using ATD that has shown that 5-HT alters delay discounting (e.g. Schweighofer et al 2008). In the present study, participants with greater 5-HT_1A_ availability discounted the value of delayed rewards to a lesser extent, leading to less impulsive choice. Although the hippocampus is typically associated with episodic memory processing and contextual learning, Peters and Büchel (2010) describe a manner in which this region may contribute to temporal discounting. These authors administered a standard discounting paradigm, but with the addition of a novel episodic condition which involved the presentation of relevant future episodes (i.e. vacation in Paris) that coincided with the later time point. They were able to show that these ‘episodic tags’ decreased participants’ discount rates and, through connectivity analyses, that this tag effect was associated with increased coupling between the ACC and the hippocampus bilaterally. Therefore, we speculate that ATD may increase delay discounting by impairing prospective memory ability through attenuated hippocampal complex activation and ACC coupling (van der Veen et al 2006); however, this hypothesis requires testing in future studies.

Whilst a relationship between hippocampal complex 5-HT_1A_ availability and performance on both tasks was observed, and despite previous research indicating that striatal 5-HT may influence decision-making (e.g. Tanaka et al 2007), we identified no significant correlations between temporal discounting and/or reward and punishment processing. This may be due in part to the fact that this region has relatively few 5-HT_1A_ receptors (Palacios et al 1990), meaning that sensitivity to observe correlations with 5-HT_1A_ availability was limited. By contrast, the hippocampal complex ROI has a high 5-HT_1A_ density (Pazos et al 1987), providing us with better sensitivity to identify correlations with the behaviour. This is mirrored by mean (SD) regional binding values within the hippocampal complex of 2.48 (0.32) compared with 0.19 (0.08) within the striatum. Therefore, our results cannot be interpreted as indicating that serotonergic transmission within the striatum is not involved in the cited decision-making tasks. However, in order to assess its involvement, it may be necessary to measure other 5-HT receptors (e.g. 5-HT_2_ receptor subtype) which are present in far greater numbers in the striatum (Joyce et al 1993).

It should also be noted that this study contained a limited sample size (*N* = 15), reducing our sensitivity to detect small effects, and an unbalanced gender distribution (two females). Whilst the correlation between 5-HT_1A_ availability and sensitivity to probability on the gambling task remained significant when the female participants were excluded, the correlation between 5-HT_1A_ availability and discount factors on the temporal discounting paradigm did not (though we note that statistical power would have been reduced due to the reduction in sample size, making this null result difficult to interpret). In order to maintain our already limited statistical power, we did not correct for the fact that we examined two ROIs and five task variables, which increases the likelihood of type I errors. Therefore, these results should be treated with caution until replicated. It will be important in future studies to include both a larger sample size and a balanced gender distribution in order to assess the impact of gender on these results.

In conclusion, we found that the influence of 5-HT on decision-making can be observed at the level of the receptor. This has implications for psychiatric disorders in which 5-HT transmission is hypothesised to be compromised, such as depression, in which abnormalities in reward/punishment processing and impulsivity have been reported (Eshel and Roiser 2010) and 5-HT_1A_ receptors may be reduced (Drevets et al 1999). It would be of great interest to use the individual differences approach we adopted in the present study to investigate whether the 5-HT_1A_ receptor is linked to poor decision-making in depression and other neuropsychiatric disorders.

## Electronic supplementary material

Below is the link to the electronic supplementary material.

Table S1 (DOCX 26 kb)

Table S2 (DOCX 25 kb)
