# Supplementary material for: The relationship between reward and punishment processing and the 5-HT1A receptor as shown by PET
Source: Psychopharmacology (Berl). 2014 Jan 16;231(13):2579–86. doi: 10.1007/s00213-013-3426-9 (PMC4057624; doi:10.1007/s00213-013-3426-9)
Supplement: Supplementary file 1 — (DOCX 26 kb) [file 213_2013_3426_MOESM1_ESM.docx]

**Supplementary Material**

**Table 1**

**Gambling Task**

| Wins | Cluster Size | *Z* Value | *X* | Y | Z | Region |
| --- | --- | --- | --- | --- | --- | --- |
| (Positive) | 22 | 3.52 | 14 | -58 | 16 | Occipital Gyrus (R) |
| (Negative) | 15 | 3.41 | -52 | -26 | -14 | Medial Temporal Gyrus (L) |
| Losses |  |  |  |  |  |  |
| (Positive)  (Positive) | 15  32 | 3.41  3.34 | 41  -54 | -62  -32 | 0  -36 | Medial Temporal Gyrus (R)  Inferior Temporal Gyrus (L) |
| (Negative) | 29 | 3.92 | -10 | -50 | 36 | Inferior Temporal Gyrus (L) |
| Probability |  |  |  |  |  |  |
| (Positive) | 55 | 4.06 | -10 | 4 | -36 | Perirhinal Cortex (L) |
| (Positive) | 292 * | 3.87 | 18 | 6 | -38 | Perirhinal Cortex (R) |
| (Positive) | 114 | 3.42 | 26 | -38 | -32 | Fusiform Gyrus (R) |

**Table 1. Summary of correlations between participants’ 5-HT_1A_ availability and sensitivities to wins, losses and probabilities. Asterisk denotes the correlation that survived small volume correction.**
