# Supplementary material for: The relationship between reward and punishment processing and the 5-HT1A receptor as shown by PET
Source: Psychopharmacology (Berl). 2014 Jan 16;231(13):2579–86. doi: 10.1007/s00213-013-3426-9 (PMC4057624; doi:10.1007/s00213-013-3426-9)
Supplement: Supplementary file 2 — (DOCX 25 kb) [file 213_2013_3426_MOESM2_ESM.docx]

**Table 2**

**Temporal Discounting**

| *K* | Cluster Size | *Z* Value | *X* | Y | Z | Region |
| --- | --- | --- | --- | --- | --- | --- |
| (Negative)  (Negative)  (Negative)  (Negative)  (Negative)  (Negative) | 411 *  23  49  31  15  16 | 4.24  4.00  3.54  3.29  3.26  3.21 | -26  -16  -8  46  26  40 | -14  -42  -88  -44  -88  -18 | -42  46  8  -34  -6  -46 | Parahippocampal gyrus (L)  Paracentral Lobule (L)  Striate Area (L)  Inferior Temporal Gyrus (R)  Occipital Gyrus (R)  Fusiform Gyrus (R) |
| *R* |  |  |  |  |  |  |
| (Positive) | 24 | 3.71 | -34 | 40 | 50 | Superior Frontal Gyrus (L) |

**Table 2. Summary of correlations between participants’ 5-HT_1A_ availability, discount rates (*K*), and utility concavities (*r*). Asterisk denotes the correlation that survived small volume correction.**
